# Determinants of Recent HIV Self-Testing Uptake Among Men Who Have Sex With Men in Jiangsu Province, China: An Online Cross-Sectional Survey

**DOI:** 10.3389/fpubh.2021.736440

**Published:** 2021-11-01

**Authors:** Jianjun Li, Gifty Marley, Ye Zhang, Yunting Chen, Weiming Tang, Yu Rongbin, Gengfeng Fu

**Affiliations:** ^1^Jiangsu Provincial Center for Disease Prevention and Control, Nanjing, China; ^2^School of Public Health, Nanjing Medical University, Nanjing, China; ^3^University of North Carolina Project-China, Guangzhou, China; ^4^Kirby Institute, University of New South Wales, Sydney, NSW, Australia; ^5^Guangdong Second Provincial General Hospital, Guangzhou, China

**Keywords:** self-testing, HIV, MSM, Jiangsu, China

## Abstract

**Background:** To help inform regarding HIV self-testing (HIVST) upscale, we assessed the determinants of recent HIVST uptake among men who have sex with men (MSM) in Jiangsu province, China.

**Methods:** We conducted a convenience online survey from March to April, 2020 among men aged ≥16 years, who had ever had sex with other men. Statistical analysis included Pearson's chi-square test, bivariate correlation, and multivariable logistic regression. *p* < 0.05 was considered statistically significant.

**Results:** Of the total 692 participants, 69.5% (481) were aged between 18 and 40 years, and 65.9% (456) had reportedly ever self-tested. Using HIVST for first HIV test (aOR = 1.98, 95% CI: 1.21–3.26), perceiving HIVST as more private (aOR = 1.41, 95% CI: 0.85–2.35), and users not needing to go to a health facility (aOR = 1.68, 95% CI: 1.20–2.34) were associated with recent HIVST as facilitating factors.

**Conclusion:** HIVST uptake rate has increased among Jiangsu MSM and can be further promoted by healthcare workers routinely recommending HIVST to their clients.

## Introduction

HIV continues to be a global public health concern regardless of the gradual decrease in HIV-related morbidity and mortality observed in the last decade (1). Irrespective of successful advances in improving the access to preventive therapy and antiretroviral therapy (ART) coverage, HIV incidence among marginalized population (such as men who have sex with men (MSM) remains on the rise globally (2). In China, MSM constituted 25.5% (*N* = 34,358) of all new HIV cases recorded in 2017 and have become the focused group of attention in most developed regions of China (3). A recent systematic review reported the prevalence of HIV among MSM in China to be 5.7% (95% CI: 5.4–6.1) with an increasing trend observed from 2001 to 2018 (4). Jiangsu province is one of the most developed provinces in the southeastern region of China. Similar to the national trends, HIV prevalence and incidence has increased among MSM in Jiangsu province over the past decade, and same sex transmission among men accounted for 54.2% (*N* = 24,592) of all new HIV cases recorded in 2020 (5, 6). According to previous studies, HIV incidence among MSM in the Jiangsu province increased from 5.10% in 2011 to 6.62% in 2015 (5).

Given the substantial burden of HIV among MSM and the unique barriers to HIV prevention faced by this susceptible population, HIV diagnosis among MSM has extensively been delayed (7). In response to the escalating HIV epidemic among MSM, China implemented comprehensive prevention strategies over the past decade. Particularly in Jiangsu province, the local government established about 350 voluntary HIV testing and counseling (VCT) centers to provide gay-friendly facility-based HIV testing services. In addition, the growing number of established MSM Community-Based Organizations (CBOs) provided peer-led HIV rapid testing and referrals for treatment. Regardless, reasons like the fear of stigma and discrimination and concerns about confidentiality continue to discourage facility-based HIV testing uptake among MSM to date. Hence, there was a need for more innovative strategies to overcome these barriers and expand HIV testing among MSM.

HIV self-testing (HIVST) is a process whereby a person who wants to know his or her HIV status collects a specimen, performs a test, and interprets the test result in private (8). The World Health Organization (WHO) recommended the strategy in 2016 after a comprehensive review of 32 randomized controlled trials (RCTs) that showed HIVST to increase HIV testing uptake (9, 10).

In addition, many studies have found HIVST acceptable to various marginalized populations (including MSM) and feasible in multiple settings (11–14). For MSM who have never tested for HIV, HIVST may represent a suitable option as it offers privacy, confidentiality, and is easy to use (15, 16). The comprehensive strategy extends an additional degree of convenience by allowing users to test in a place and at a time that they feel comfortable. In addition, HIVST being a cost-effective intervention could reduce the financial burden of seeking HIV testing services in health facilities and the cost of national HIV intervention (17–19).

To date, HIVST has invariably played an influential role in helping reach hidden MSM with HIV testing services in China since its introduction (20). The strategy fulfilled a prominent role in keeping HIV testing services available during the COVID-19 epidemic when access to facility-based HIV testing was impossible (21–23).

However, upscaling HIVST to the national level is delayed due to social barriers hindering HIVST uptake. In addition, published studies that access the uptake rate of HIVST among MSM in Jiangsu province are uncommon to date. Therefore, our study aimed to objectively assess current HIVST uptake among MSM in Jiangsu province, China, and identify the barriers and facilitators promoting scaling up HIVST.

## Methods

### Study Design and Participant Recruitment

A convenience online survey was conducted between March and April, 2020 to evaluate MSM social demographic characteristics, sexual and HIV testing history, and attitude toward HIVST in Jiangsu, China. The questionnaire was created by our study group and revised by focus group discussion with key opinion leaders (KOL) and volunteers from local MSM community. Links to the online survey were promoted *via* local MSM-led CBOs and on social media (WeChat and QQ). The criteria for eligible participants included those born biologically male, at least 16 years of age, had ever tested for HIV, and had ever had anal sex with a man during the last 6 months. Interested participants were asked to click the link and then directed to the survey eligibility screening page, which was hosted on wjx.cn. Participants meeting the eligibility criteria were directed to an informed consent page before beginning the survey where data was collected.

### Data Collection

Socio-demographic characteristics included age, marital status, highest education level, monthly income, sexual orientation, and disclosure of sexual orientation to others apart from sexual partners and healthcare workers. Sexual and HIV testing history variables included number of sexual partners, condom use rate, HIV testing frequency in last 6 months, and HIV status.

HIV testing variables included HIVST experiences, facilitators, and barriers to HIVST including privacy, operation, and cost, which were analyzed with the help of a real-time test reader which could recognize and send result interpretation to testers. Participants also had to report if they had ever self-tested for HIV, the number of times they self-tested last year, the means of acquiring HIVST kits, whether they used HIVST for first-ever HIV test, if they had ever been administered (given) an HIVST by other people, if they had ever had a positive result in any self-test(s), and if they had ever considered using an HIVST kit to test a sex partner before having sex.

Furthermore, we evaluated the attitudes toward HIVST by asking the participants about the comfortable ways to report HIVST results to others (including healthcare workers), preferred means of reporting HIVST results, anxiety or concerns regarding HIVST, confidence in coping with HIVST results, and recommendations that could encourage them to opt for HIVST.

### Measures

We defined HIVST as an individual administering an HIV test to himself and interpreting the result in private, rather than at a facility. Recent HIVST referred to having self-tested at least once in the preceding year. We defined the HIVST uptake rate as the proportion of our study participants that had ever self-tested before the study. We categorized participants who had previously used HIVST kits as ever self-tested, and participants who had never used HIVST kits as never self-tested.

### Data Analysis

We descriptively summarized the socio-demographic characteristics, sexual behaviors, and HIV testing history of the participants, by comparing those who had ever self-tested with those who had never self-tested. A descriptive analysis was also conducted to summarize the attitudes of the participants toward HIVST by a group. Pearson's chi-square test was used to test for statistically significant differences in opinions and beliefs between the two groups about the importance of HIVST. Logistic regression was used to investigate associations between socio-demographic and behavioral variables, and HIVST (self-testers vs. non-self-testers). Variables found to be marginally associated (a priori determined as *p* < 0.20) with HIVST uptake in crude bivariate analysis were included in a multivariable logistic regression model. Statistical significance was defined as *p* < 0.05. All analyses were performed using SPSS software (IBM SPSS statistics version 20.0.0).

## Results

Overall, 716 participants clicked the link and initiated the survey, but we excluded 24 participants for various reasons (12 were women, nine were under 16 years old, and three reported no anal sex with a man in the last 6 months).

### Socio-Demographic Characteristics and Sexual Behaviors

Overall, the majority of participants (53.3%, 369) were in the age group of 26–40 years, 30.5% (211) were aged over 40 years, and 16.2% (112) were aged 25 years and below. Most of the participants (67.5%, 467) self-identified as gay, 51.3% (355) had a college or university degree, and 55.2% (382) had a monthly income over 6,000 RMB. Among the participants, 49.1% (340) were single, 79.8% (552) had disclosed their sexual orientation to others apart from their sexual partners, and the majority of them had met their sexual partners through online platforms (85.8%, 594). Many participants (63.2%, 437/692) had at least one regular sexual partner, and few (26%, 180/692) reported having multiple temporary sexual partners in the preceding 6 months. Unprotected anal intercourse was more frequent among MSM with their regular partners than with casual partners in the last 6 months (26.6 vs. 10.8%). Table 1 further summarizes the socio-demographic and sexual behavioral characteristics of the participants.

**Table 1:** Descriptive summary of participants' socio demographic characteristics and sexual behaviors.

**Variables**	**Total**	**HIVST experience**		***p***
	***n* = 692 (%)**	**Ever**	**Never**	
		***n* = 456 (%)**	***n* = 236 (%)**	
**Age**				0.198
<25	112 (16.2)	82 (18.0)	30 (12.7)	
26–30	185 (26.7)	116 (25.4)	69 (29.2)	
31–40	184 (26.6)	127 (27.9)	57 (24.2)	
41–50	155 (22.4)	98 (21.5)	57 (24.2)	
>50	56 (8.1)	33 (7.2)	23 (9.7)	0.628
**Marital status**				
Single	340 (49.1)	227 (49.8)	113 (47.9)	
Married	239 (34.5)	152 (33.3)	87 (36.9)	
Divorced/separated	113 (16.3)	77 (16.9)	36 (15.3)	
**Highest education level**				0.696
High school	295 (42.6)	199 (43.6)	96 (40.7)	
Some college or university	355 (51.3)	231 (50.7)	124 (52.5)	
Post Graduate	42 (6.1)	26 (5.7)	16 (6.8)	
**Monthly income**				0.388
<1500 RMB	28 (4.0)	20 (4.4)	8 (3.4)	
1500 – 3000RMB	142 (20.5)	98 (21.5)	44 (18.6)	
3,001–5,000 RMB	140 (20.2)	111 (24.3)	59 (25.0)	
5,001–8,000RMB	212 (30.6)	144 (31.6)	68 (28.8)	
>8,000RMB	170 (24.6)	83 (18.2)	57 (24.2)	
**Sexual identity**				0.583
Homosexual	467 (67.5)	302 (66.2)	165 (69.9)	
Bisexual	211 (30.5)	145 (31.8)	66 (28.0)	
Unsure	14 (2.0)	9 (2.0)	5 (2.1)	
**Ever disclosed sexual orientation or history to others aside sexual partners**				<0.001^*^
Yes	483 (69.8)	373 (81.8)	110 (46.6)	
No	209 (30.2)	83 (18.2)	126 (53.4)	
**Ever disclosed sexual orientation or history to healthcare worker**				<0.001^*^
Yes	359 (51.9)	268 (58.8)	91 (38.6)	
No	333 (48.1)	188 (41.2)	145 (61.4)	
**No. of sexual partners in last 6 months**				<0.001^*^
0	98 (14.2)	49 (10.7)	49 (20.8)	
1	298 (43.1)	169 (37.1)	129 (54.7)	
≥2	296 (42.8)	238 (52.2)	58 (24.6)	
**Condom use rate with regular partner in the last 6 months**				<0.001^*^
Never	35 (8.0)	35 (10.6)	0 (0.0)	
Sometimes	149 (34.1)	100 (30.4)	49 (45.4)	
Always	253 (57.9)	194 (59.0)	59 (54.6)	
**Condom use rate with casual partner in the last 6 months**				0.006^*^
Never	0 (0.0)	0 (0.0)	0 (0.0)	
Sometimes	75 (28.4)	44 (23.5)	31 (40.3)	
Always	189 (71.6)	143 (76.5)	46 (59.7)	
**How often do you test for HIV**				<0.001^*^
Every 3 month	398 (57.5)	309 (67.8)	89 (37.7)	
Every 6 months	140 (20.2)	84 (18.4)	56 (23.7)	
1year and above	154 (22.3)	63 (13.8)	91 (38.6)	
**Number of times tested for HIV last year**				<0.001^*^
Never	80 (11.6)	27 (5.9)	53 (22.5)	
Once	117 (16.9)	54 (11.8)	63 (26.7)	
Twice	176 (25.4)	129 (28.3)	47 (19.9)	
Thrice	125 (18.1)	83 (18.2)	42 (17.8)	
4times and above	194 (28.0)	163 (35.7)	31 (13.1)	
**Know your HIV status**				0.221
Yes	642 (92.8)	427 (93.6)	215 (91.1)	
No	50 (7.2)	29 (6.4)	21 (8.9)	
**HIV status**				0.470
Positive	102 (15.9)	71 (16.6)	31 (14.4)	
Negative	540 (84.1)	356 (83.4)	184 (85.6)	

* *p < 0.001, RMB = Chinese Renminbi*.

### HIV Testing and Self-Testing Experience

Of the 692 participants, 65.9% (456) had ever self-tested for HIV and 15.9% (102) were reportedly living with HIV (PLWH). Many participants (67.8% self-testers and 37.7% non-self-testers) tested for HIV every 3 months. Most participants (71.5%, 495) tested for HIV more than twice in the preceding year, of which 65.9% (456) had ever self-tested, and 42.5% (194) used HIVST for their first-ever HIV test. The majority of the participants (81%, 560) had also considered home testing with a partner right before sex.

Among the 59.6% (365/612) participants that self-tested in the preceding year, 77.8% (284/365) of them self-tested more than twice. In addition, among 69.6% (71/102) of PLWH who had ever self-tested, 76.1% (54/71) reportedly used HIVST for their first-ever HIV test and obtained reactive results. Among the PWLH, 52.9% (54/102) of all positives discovered their status through HIVST. A total of 41.9% (191) of ever self-tested participants bought HIVST kits online, 36.6% (167) obtained the kits from implementation programs, and 33.6% (153) got tested by a friend (including a sexual partner). About half of the participants (58.8%) received an HIV self-test from other people, and 11.8% (54/456) obtained reactive results in at least one of their HIVSTs. In addition, 42.5% (194) of participants used the HIVST kit for their first-ever HIV test. Further details on the self-testing history of the participants are summarized in Table 2.

**Table 2:** HIV self-testing experience among MSM in Jiangsu province, China, 2020.

**Variables**	**Total [%(*n*/*N*)]**
Ever self-tested for HIV	65.9 (456/692)
Tested for HIV at least once last year	88.4 (612/692)
Self-tested for HIV last year	
Never	47.3 (327/692)
Once	11.7 (81/692)
Twice	22.4 (155/692)
Thrice	9.2 (64/692)
4times and above	9.4 (65/692)
Number of those tested last year who used HIVST	59.6 (365/612)
Tested for HIV once last year using HIVST	30.8 (36/117)
Tested for HIV twice last year using HIVST	73.3 (129/176)
Tested for HIV more than twice using HIVST	62.7 (200/319)
Where did you obtain your HIV self-test kit	
Bought online	41.9 (191/692)
Participated in research projects	36.6 (167/692)
From a friend (including a sexual partner)	33.6 (153/692)
Other means	24.3 (111/692)
Pharmacy	5.5 (25/692)
Used HIVST kit for first HIV test	42.5 (194/692)
Ever been administered (given) an HIV self-test from other people	58.8 (407/692)
Ever had a positive result in any of your HIV self-test(s)	
Yes	11.8 (54/456)
No	85.2 (389/456)
Could not read result	2.9 (13/456)
Number of PLWH who had ever used HIVST	69.6 (71/102)
Number of PLWH who used HIVST for first HIV test	76.1 (54/71)
Number of PLWH who obtained reactive results in HIVST	76.1 (54/71)
Number of PLWH reactive HIVST confirmed positive at a clinic or CDC	100 (54/54)
Total number of positives identified through HIVST	52.9 (54/102)

*CDC, Center for Disease Control and prevention; PLWH, Persons living with HIV; HIVST, HIV self-testing*.

### Reporting of HIV Self-Testing Results

Table 3 shows the distinctions in opinions and beliefs about the importance of HIVST among ever self-tested and never self-tested participants. Among 456 ever self-tested participants, 53.1% were most comfortable sharing their HIVST result with a researcher/healthcare worker in person, while 35.7% were comfortable using an automated test reader. Never self-tested participants were comfortable using an electronic test reader (45.8%) and reporting in person (41.9%). The majority of self-testers (60.1%, 274) preferred reporting HIVST results by sending a picture of the used self-test kit, whereas 44.1% preferred to submit the results in person. Among the participants, 60.1% (274) self-testers and 40.7% (96) non-self-testers used a home HIV test kit to test a sexual partner before having sex. Few self-testers (32.5%, 148) and non-self-testers (23.7%, 56) were anxious about waiting for their HIVST results, while more number of never self-tested participants (50.0%) compared to self-testers were anxious about understanding their HIVST results. Ever self-tested participants were much more confident than never self-tested participants about coping with waiting for a few days for results from a clinic (83.3 vs. 69.5%), waiting 15–20 mins for HIVST results (69.3 vs. 54.2%), and getting a reactive HIVST result (67.8 vs. 46.6%). The majority of never self-tested participants (84.3%, 199) were likely to use HIVST if recommended by a healthcare provider and a sexual partner (69.5%, 164).

**Table 3:** Disparities of opinions and belief about HIV self-testing importance between participants whoever self-tested for HIV or not.

**Variables**	**Total**	**HIVST experience**		***p***
	***n* = 692 (%)**	**Ever**	**Never**	
		***n* = 456 (%)**	***n* = 236 (%)**	
**How comfortable would you feel sharing your HIV self-test result with a researcher/health worker?**				
In person	341 (49.3)	242 (53.1)	99 (41.9)	0.006^*^
By using a real-time test reader	271 (39.2)	163 (35.7)	108 (45.8)	0.010^*^
By mailing in your used self-test swab to health worker	247 (35.7)	159 (34.9)	88 (37.3)	0.529
By using social media	204 (29.5)	154 (33.8)	50 (21.2)	0.001^*^
By text	202 (29.2)	162 (35.5)	40 (16.9)	0.000^*^
By email	159 (23.0)	98 (21.5)	61 (25.8)	0.197
**How would you MOST prefer to share your HIV self-test result with a researcher?**				
By taking and sending a picture of your used self-test swab	323 (46.7)	274 (60.1)	49 (20.8)	<0.001^*^
By phone	313 (45.2)	218 (47.8)	95 (40.3)	0.058
In person	273 (39.5)	169 (37.1)	104 (44.1)	0.074
Via text message	264 (38.2)	188 (41.2)	76 (32.2)	0.021^*^
By email	172 (24.9)	101 (22.1)	71 (30.1)	0.022^*^
By mailing in your used self-test swab	167 (24.1)	79 (17.3)	88 (37.3)	<0.001^*^
In writing (e.g., checking a box on a postcard and mailing it)	43 (6.2)	24 (5.3)	19 (8.1)	0.150
Do not share	14 (2.0)	14 (3.1)	0 (0)	0.007^*^
**How likely is it that you would use a home HIV test kit to test a sex partner before having sex?**				<0.001^*^
Unlikely	81 (11.7)	53 (11.6)	28 (11.9)	
Not sure	241 (34.8)	129 (28.3)	112 (47.5)	
Likely	370 (53.5)	274 (60.1)	96 (40.7)	
**You might be anxious or concerned about**				
Using the test correctly when you start using home HIV tests	237 (34.2)	147 (32.2)	90 (38.1)	0.121
Waiting for the results to appear when you start using home HIV tests	204 (29.5)	148 (32.5)	56 (23.7)	0.017^*^
The accuracy of the results when you start using home HIV tests	311 (44.9)	201 (44.1)	110 (46.6)	0.526
Understanding the result when you start using home HIV tests	276 (39.9)	158 (34.6)	118 (50.0)	<0.001^*^
**You are very confident that you could cope with**				
Waiting for a few days for my HIV test results from a clinic	544 (78.6)	380 (83.3)	164 (69.5)	<0.001^*^
Being diagnosed with HIV	427 (61.7)	256 (56.1)	171 (72.5)	<0.001^*^
Waiting 15–20 mins for the results of a home HIV test that you did yourself	444 (64.2)	316 (69.3)	128 (54.2)	<0.001^*^
Getting a reactive result when using a home HIV test	419 (60.5)	309 (67.8)	110 (46.6)	<0.001^*^
**You are likely to use self-testing if**				
Recommended by your healthcare provider	595 (86.0)	396 (86.8)	199 (84.3)	0.012^*^
Recommended by your sexual partner	561 (81.1)	397 (87.1)	164 (69.5)	<0.001^*^
Tested for other STIs	556 (80.3)	392 (86.0)	164 (69.5)	<0.001^*^
Recommended by Government and advertised on TV as well as social media	385 (55.6)	258 (56.6)	127 (53.8)	0.654
Recommended or offered by my friends and colleagues (classmates, work colleagues, family friends)	420 (60.7)	295 (64.7)	125 (53.0)	<0.001^*^

*STIs, sexually transmitted infections*.

* *p < 0.001*.

### Factors Associated With Recent HIVST Uptake

In adjusted logistic regression shown in Table 4, participants who had a regular sexual partner in the last 6 months were less likely to have recently self-tested (aOR = 0.61, 95% CI: 0.43–0.86). Also, having obtained HIVST kits from a research project (aOR = 0.43, 95% CI: 0.24–0.77) was associated with decreased odds of recent HIVST. However, using HIVST for first-ever HIV testing (aOR = 1.98, 95% CI 1.21–3.26) and prefer HIVST as no needing to go to a hospital or clinic (aOR = 1.68, 95% CI 1.20–2.34) were positively associated with recent HIVST uptake. None of the socio-demographic characteristics were associated with recent HIVST among MSM participants.

**Table 4:** Factors associated with recent HIVST (self-testing at least once last year) among MSM in Jiangsu province, China 2020.

**Variables**	**OR (95% C.I)**	***p***	**aOR (95% CI)**	***p***
**Age (years)**				
<25	1			
26–40	0.75 (0.49–1.15)	0.189		
>40	0.57 (0.36–0.91)	0.019		
**Have had a regular sexual partner in the last 6months**				
No	1	–	1	–
Yes	0.62 (0.44–0.87)	0.006	0.61 (0.43–0.86)	0.004
**UAI with regular partner in last 6months**				
No	1	–	1	–
Yes	0.66 (0.45–0.97)	0.036	0.72 (0.40–1.31)	0.280
**Obtained HIVST kits in a research project**				
No	1	–	1	–
Yes	0.46 (0.29–0.73)	0.001	0.43 (0.24–0.77)	0.004
**Obtained HIVST kits from other means (PE, partner, etc.)**				
No	1	–	1	–
Yes	1.16 (0.79–1.88)	0.541	0.57 (0.32–1.03)	0.062
**Used HIVST in first ever HIV test**				
No	1	–	1	–
Yes	2.00 (1.22–3.28)	0.006	1.98 (1.21–3.26)	0.007
**HIVST is more private**				
No	1	–	1	–
Yes	1.63 (1.00–2.66)	0.049	1.41 (0.85–2.35)	0.182
**HIVST does not require one to go to a hospital or clinic**				
No	1	–	1	–
Yes	1.69 (1.23–2.31)	0.001	1.68 (1.20–2.34)	0.002
**No expert guidance on site**				
No	1	–	1	–
Yes	1.47 (1.08-1.96)	0.013	1.52 (1.11–2.08)	0.010
**Cost of purchase too expensive**				
No	1	–	1	–
Yes	0.66 (0.42–1.04)	0.072	0.66 (0.41–1.06)	0.085
**HIVST has no disadvantages**				
No	1	–	1	–
Yes	1.71 (1.08–2.71)	0.023	1.98 (1.22–3.21)	0.006

*NB: OR, odds ratio; aOR, adjusted odds ratio (each variable was adjusted for age); PE, peer educator; UAI, unprotected anal intercourse*.

*p-value significant at <0.05*.

### Facilitators and Barriers of HIVST

Privacy (89.3%), convenience (68.1%), ability to self-test (63.3%), the opportunity for partner testing at the same time (49.9%), and being less embarrassing (47.1%) were the top five facilitators for HIVST uptake among all participants. Alternatively, the absence of additional sexual healthcare services (48.7%), no professional guide (43.8%), fear of less accurate result (38.4%), cost of kits (27.9%), and difficulty in performing testing (19.2%) were the main barriers cited (Table 5).

**Table 5:** Facilitators and barriers to HIVST uptake among MSM in Jiangsu province of China, 2020.

**Variables**	**Total [*n* = 692(%)]**
**Facilitators of HIV self-testing**	
More private	618 (89.3)
More convenient	471 (68.1)
I could test myself	438 (63.3)
Opportunity to test partner(s) at the same time	345 (49.9)
Less embarrassing	326 (47.1)
Allows me to test when I want	325 (47)
Saves time	320 (46.2)
No need to go to a doctor or clinic	251 (36.3)
Does not require blood to be taken	216 (31.2)
Gives results in 15–20 mins	214 (30.9)
**Barriers to HIV self-testing**	
Not possible to have a full sexual health check at the same time	337 (48.7)
No health professional present when I tested	303 (43.8)
Results are less accurate	266 (38.4)
I need to buy the kits by myself	193 (27.9)
Difficult to perform	133 (19.2)
It is expensive	89 (12.9)
Being alone when I tested myself	89 (12.9)
There was nothing I didn't like	89 (12.9)
Waiting 15–20 mins for results	33 (4.8)
Other	13 (1.9)

## Discussion

HIV self-testing could effectively expand HIV testing and promote HIV case identification. This study extends the present literature by analyzing the facilitators and barriers of HIVST and explores the driving forces of recent HIVST uptake among MSM in Jiangsu province. In our study, only 65.9% of the participants had ever self-tested, while 80% had self-tested at least once recently (in the preceding year). We also noted that having used HIVST for the first-ever HIV test was positively associated with recent self-testing. Furthermore, not needing to go to a facility for HIVST increased the odds of recent HIVST. However, having a regular partner in the preceding 6 months decreased the odds of recent HIVST. Privacy, convenience, and the ability to self-test were the key driving factors of HIVST uptake, while being unable to have a full sexual health check-up and the presence of a healthcare worker during HIVST remained barriers. HIVST is nevertheless a vital strategy for increasing HIV testing coverage, and there is an urgent need to further promote HIVST among MSM.

Though HIVST uptake rate among MSM in Jiangsu province has improved, there is still room for further improvement. According to our findings, only 65.9% of MSM in our study had ever self-tested for HIV. Our observed rate is higher than the rates found in a study conducted in the province in 2014 (26.2%) (24), and in recent studies conducted in Brazil (49.1%) (13) and China (25). Considering the limitations of facility-based testing and the impact of COVID-19, upscaling HIVST uptake among Chinese MSM will require more efforts. We speculate that the combined effects of various governments and CBO-led HIVST intervention projects accounted for this increase and could improve rates with additional strategies. For example, projects could use crowdsourcing to enhance the impact of HIVST interventions, as it had increased HIVST uptake among MSM by almost two-folds in a recent study (26). Also, intervention programs should expand digital and secondary distribution methods as they have been proven effective in reaching hidden MSM (27, 28). In addition, the government should consider adopting digital and cellular reporting systems to facilitate timely reporting of results and linkage to post-HIVST care (29–31). Moreover, the country needs policies to standardize HIVST delivery and reporting systems before upscaling HIVST for a nationwide rollout (20).

In addition, HIVST increased HIV testing frequency among MSM. Similar to findings of previous studies (32), we found that ever self-tested MSM reported higher rates of recent HIV testing than never self-tested participants. We also observed that the majority of the participants who frequently tested for HIV (two or more times per year) used HIVST. Our findings further conform to the findings of a recent study in which HIVST was found to significantly increase the frequency of bi-annual HIV testing from 337.8% before the study to 84.5% among South African MSM (33). The findings of a systematic review also support this view with their conclusion that HIVST could increase HIV testing frequency and reach first-time HIV testing among MSM (34). Accordingly, access to HIVST kits should be improved using online service platforms, secondary distribution, and partner testing interventions to improve the rate of routine HIV testing among MSM.

HIV self-testing could also facilitate early HIV diagnosis, as we found that the majority of PLWH in our study (76.1%) first discovered their status through HIVST. This observation is higher than the 3.5% of positives identified by HIVST among a population of MSM who had never tested for HIV in a previous study (30). Thus, HIVST could be vital for the timely diagnosis in MSM with high sexual risk behaviors, who might refuse facility-based testing. The strategy could also save money for individuals residing in remote places and reduce the national investment in HIV prevention interventions. Hence, there is a more urgent need to upscale HIVST in China to the national level.

Furthermore, we found that MSM who used HIVST for their first-ever HIV test were more likely to use HIVST often. The theory of health belief, which suggests that perceived benefits in the utilization of health services influence the attitude and continued use of the services to form a behavior, could explain this observation (35). Therefore, we believe that the benefits for users of HIVST, which include the ease of use, convenience, and privacy, may have contributed to this observation. However, we also found that having a regular partner reduced the odds of recent HIVST among MSM. MSM in committed relationships or those with a regular partner feel a decreased sense of being at risk of HIV infection due to trust. It also implies that MSM who consider HIVST with casual partners before sex perceive higher risk of exposure to HIV infection and the need to test (36). We consider partner testing before sex a strategic way to assess risk, and recommend that it should be encouraged by ensuring unrestricted access to HIVST kits at multiple levels. Therefore, the government and CBOs should focus more on HIVST promotion at vantage points where casual hookups are more likely to occur (including online platforms). HIVST promotion interventions should also highlight the benefits of couple testing for MSM to encourage routine testing among MSM with regular partners. In addition, we require policies to enable healthcare workers to offer or recommend HIVST to their MSM clients as part of the routine services.

Notably, low self-efficacy persists as a barrier to HIVST uptake. In our study, only 34.2% of MSM participants were confident about conducting HIVST correctly, while 39.9% reported they might be anxious about interpreting HIVST results accurately. Unfortunately, a previous study conducted in China made similar observations when the study participants cited anxiety regarding conducting HIVST in an incorrect manner as a barrier to its use (24). Nonetheless, these barriers can be mitigated by improving the knowledge on HIVST among MSM, enhancing accessibility to HIVST procedures, and providing assistance services, especially to never self-tested MSM. Therefore, we recommend that HIVST kit retailers and distributors should provide detailed practical guidelines on HIVST kit operation as part of pre-test counseling. In addition, test kit developers should consider making pictorial HIVST guidelines freely available online for easy reference by users. Online video tutorials in local dialects explaining HIVST procedures are also needed for easy referencing. Also, healthcare providers should utilize the existing cellular and digital platforms to extend supervision to HIVST users. Furthermore, researchers should investigate automated systems that are capable of ascertaining test accuracy and reading rapid test results for future use.

Overall, our study findings have implications for nationwide HIVST promotion and upscale. Considering the increasing trend in HIVST uptake rate among MSM in China, it is imperative for the government to consider the development of HIVST-related technologies to resolve barriers linked to care and eliminate errors by users. This is very important as facilitating the development of a standard, simple, and accurate result interpretation system will better promote the utilization of HIVST. In addition, with the availability of 5G, online-operation-guides, and artificial intelligence (AI) systems, the adoption of digital media technology to compliment pre- and post-HIVST counseling efforts could further ease HIVST services for MSM.

This study has several limitations. First, the survey captured a convenient sample of MSM online in the Jiangsu province of China through a cross-sectional study design, which implied that the study participants were younger and more active on the internet. Consequently, our findings may not accurately represent the Chinese MSM population in Jiangsu. Second, all the results from data analysis were based on self-reported information, which may suffer from information bias. However, since computer-based surveys are more convenient for collecting sensitive information, we believe the information bias may not have a significant impact on the current study. Third, we did not collect data about the acceptability of HIVST compared to facility-based testing among never-tested MSM, which could have presented an added advantage for the promotion of HIVST among harder-to-reach MSM. Also, the impact of using HIVST for first-ever HIV test on recent HIVST uptake may be over- or under-estimated. Hence, this finding should be interpreted with caution. Future studies should also seek to review and access the direct impact of using HIVST for first-ever HIV test and recent HIVST.

## Conclusion

Our findings show that HIVST has surpassed facility-based testing and continues to play an essential role in reaching key populations. Though HIVST uptake among Jiangsu MSM has increased, further promotion is needed to increase the reach. As such, MSM who visit healthcare facilities should be encouraged to help in distributing HIVST kits to their intimate partners and peers. Additionally, healthcare providers should provide more information and recommend HIVST to their key client population. There is also a need for more propaganda about HIVST accuracy and availability in China and innovative ways to improve the reading of test results for users. Executing these approaches will help to expand HIVST uptake among MSM in Jiangsu province.

## Data Availability Statement

The raw data supporting the conclusions of this article will be made available by the authors, without undue reservation.

## Ethics Statement

The studies involving human participants were reviewed and approved by the Institutional Review Boards of Jiangsu Provincial Centre for Disease Control and Prevention (Project Number: JSJK2019-B016-03). The patients/participants provided their written informed consent to participate in this study.

## Author Contributions

JL, GM, YZ, WT, and GF designed the study, interpreted the data, and wrote the manuscript. JL and YC carried out the online survey and analyzed the data. YR supervised the study. All authors listed have approved it for publication.

## Funding

This study was supported by the National Key Research and Development Program of China (2017YFE0103800), the National Institutes of Health (R34MH119963), National Science and Technology Major Project (2018ZX10101-001-001-003), National Natural Science Foundation of China (81903371), and the National Social Science Fund of China (No. 19CSH018).

## Conflict of Interest

The authors declare that the research was conducted in the absence of any commercial or financial relationships that could be construed as a potential conflict of interest.

## Publisher's Note

All claims expressed in this article are solely those of the authors and do not necessarily represent those of their affiliated organizations, or those of the publisher, the editors and the reviewers. Any product that may be evaluated in this article, or claim that may be made by its manufacturer, is not guaranteed or endorsed by the publisher.
